# VANTED: A system for advanced data analysis and visualization in the context of biological networks

**DOI:** 10.1186/1471-2105-7-109

**Published:** 2006-03-06

**Authors:** Björn H Junker, Christian Klukas, Falk Schreiber

**Affiliations:** 1 Department of Molecular Genetics, Leibniz Institute of Plant Genetics and Crop Plant Research (IPK), Corrensstr. 3, 06466 Gatersleben, Germany

## Abstract

**Background:**

Recent advances with high-throughput methods in life-science research have increased the need for automatized data analysis and visual exploration techniques. Sophisticated bioinformatics tools are essential to deduct biologically meaningful interpretations from the large amount of experimental data, and help to understand biological processes.

**Results:**

We present VANTED, a tool for the visualization and analysis of networks with related experimental data. Data from large-scale biochemical experiments is uploaded into the software via a Microsoft Excel-based form. Then it can be mapped on a network that is either drawn with the tool itself, downloaded from the KEGG Pathway database, or imported using standard network exchange formats. Transcript, enzyme, and metabolite data can be presented in the context of their underlying networks, e. g. metabolic pathways or classification hierarchies. Visualization and navigation methods support the visual exploration of the data-enriched networks. Statistical methods allow analysis and comparison of multiple data sets such as different developmental stages or genetically different lines. Correlation networks can be automatically generated from the data and substances can be clustered according to similar behavior over time. As examples, metabolite profiling and enzyme activity data sets have been visualized in different metabolic maps, correlation networks have been generated and similar time patterns detected. Some relationships between different metabolites were discovered which are in close accordance with the literature.

**Conclusion:**

VANTED greatly helps researchers in the analysis and interpretation of biochemical data, and thus is a useful tool for modern biological research. VANTED as a Java Web Start Application including a user guide and example data sets is available free of charge at .

## Background

In the last few years the methodology of biochemical research has undergone tremendous changes. Various massively-parallel techniques have been developed, generating ever-increasing amounts of experimental data, from which a top-down view of the biochemistry of an organism is made possible. These methods include metabolite profiling [1,2], transcript profiling [3,4], and automatized enzyme assays [5]. The interpretation of the data is usually limited by analysis and visualization procedures. The central task of data visualization is to bring large amounts of data into a form that shows the data with reasonable precision, while at the same time being readable and understandable. Often the data generated by the methods described above is presented in complex tables that do not include additional biological information such as the network structure of underlying biological processes.

Several tools have been developed to represent, visualize, and analyze biological networks and data. Initially there were databases such as KEGG [6] that store information about the structure of metabolic networks. Secondly, tools for the visualization of biological networks were developed [7-9]. The third generation consists of tools to visualize experimental data in the network context [10-13]. Most of these data visualization tools follow a similar procedure: as a source for the networks they either rely on a built-in pathway collection or they make use of publicly available pathway databases. In some of the tools it is possible to edit and layout the networks. Then, imported experimental data is mapped onto the network, in most cases by applying a false color code to the nodes or edges of the network according to observed changes between two experiments. This kind of colored map is also called a heatmap. The mapping of experimental data is often restricted to expression data, the mapping of metabolite data is rarely also supported. Some of the tools additionally allow statistical analysis of the data. Examples of such data visualization tools are Cytoscape [10], MapMan [11], KaPPA-View [12], PathwayExplorer [13], and probably most prominently the Omics Viewer included in MetaCyc-related databases [14] such as AraCyc [15]. A detailed description of these tools is given in the Discussion section. However, with the exception of PathwayExplorer [13], none of these tools support the direct comparison of more than two data sets with each other, for example data from different transgenic lines or time series. Furthermore, several data visualization tools rely on static maps, which means the data is mapped onto pictures which cannot be modified by the user or dynamically changed depending on database entries.

To address the restrictions in existing systems we developed VANTED, a tool for the visualization and analysis of networks with related experimental data. It is the extended stand-alone successor of the prototypic data exploration module of the DBE-information system [16]. VANTED is designed to help scientists with the interpretation of large-scale biochemical data sets. It allows the import of any type of biochemical data (e. g. transcript, protein, metabolite) from different growth conditions and time-points, network loading and editing, and the mapping of the data on the corresponding dynamic networks (i. e., pathways). The system offers a variety of new functionalities for visual exploration, statistical calculations (*t*-test, outlier identification, correlation analysis), data clustering with self-organizing maps, and more. VANTED is a Java Web Start application and thus platform-independent. It is available free of charge. 

The remainder of the paper is structured as follows. First the VANTED system is described in the Implementation section. Then we will discuss the main features of VANTED, which was used to visualize recently published mid-scale biochemical data sets. Finally new biological insights are discussed and the system is compared to existing tools.

## Implementation

VANTED is based on the extensible graph library and editor Gravisto [17]. Gravisto is a system which follows the Model-View-Controller (MVC) paradigm. It is designed to be extensible via a plugin mechanism. VANTED is implemented in Java and is therefore platform-independent. The application uses the Java Web Start technology for easy installation and automatic updates. As an alternative a Windows setup file is provided for situations where the application needs to be used on computers with no internet connection.

The system is extensible with Java scripts (using BeanShell [18]) and Ruby scripts (using JRuby [19]). This enables the user to dynamically extend VANTED with new algorithms for analysis, graph layout, data exchange, and other functionalities. Example scripts as well as documentation for this functionality are available from the VANTED website.

In the following subsections we briefly explain the methods used in VANTED. More detailed descriptions can be found in the user's guide available from the VANTED website.

### Visualization

The basic graph visualization routines for displaying and layouting graphs in VANTED are based on the underlying Gravisto implementation [17]. With additional view plugins and by using the JFreeChart library [20] the display of experimental data in the graph view is made possible.

### Statistical tests

The Student's *t*-test and the Welch-Satterthwaite *t*-test are implemented by using the Jakarta Mathematics Library [21]. The nonparametric *U *-test (Wilcoxon, Mann-Whitney test) is implemented according to [22]. The David quick-test for normal distribution and the Grubbs' test for outliers are performed as detailed in [23].

### Correlation analysis

For the calculation of Pearson's product-moment or Spearman's rank correlation coefficient a list of value pairs needs to be extracted from the data set. A value pair between two substances to be correlated is created if three annotations correspond: (1) the plant/genotype name, (2) the time value (if present), (3) the replicate number. The result of these lookup and filter operations are two lists of values (for the Spearman correlation coefficient these values are exchanged by rank values). The significance of a particular correlation factor is checked with an approximation to the Student distribution [22].

### Self-organizing maps for the clustering of time series data

For the self-organizing map (SOM) algorithm first a training phase is performed in which clusters of common input patterns in the data are identified. Secondly, a lookup phase assigns each input vector to the best fitting cluster. The principle of the SOM-algorithm is described in [24]. In the following the data preparation as well as the processing of the algorithm results are outlined.

The training phase as well as the lookup-phase make use of normalized input vectors, which are created from an ordered set of average sample values. The ordering is determined by the superset of covered time points for all measured substances. During the lookup phase target clusters are determined by the minimum distance between the input vectors and the model vectors which are part of the SOM. Further layout-, filter-, and coloring-operations on the clustered graph nodes are then possible.

## Results

### Summary of VANTED's features

**Figure 1:**
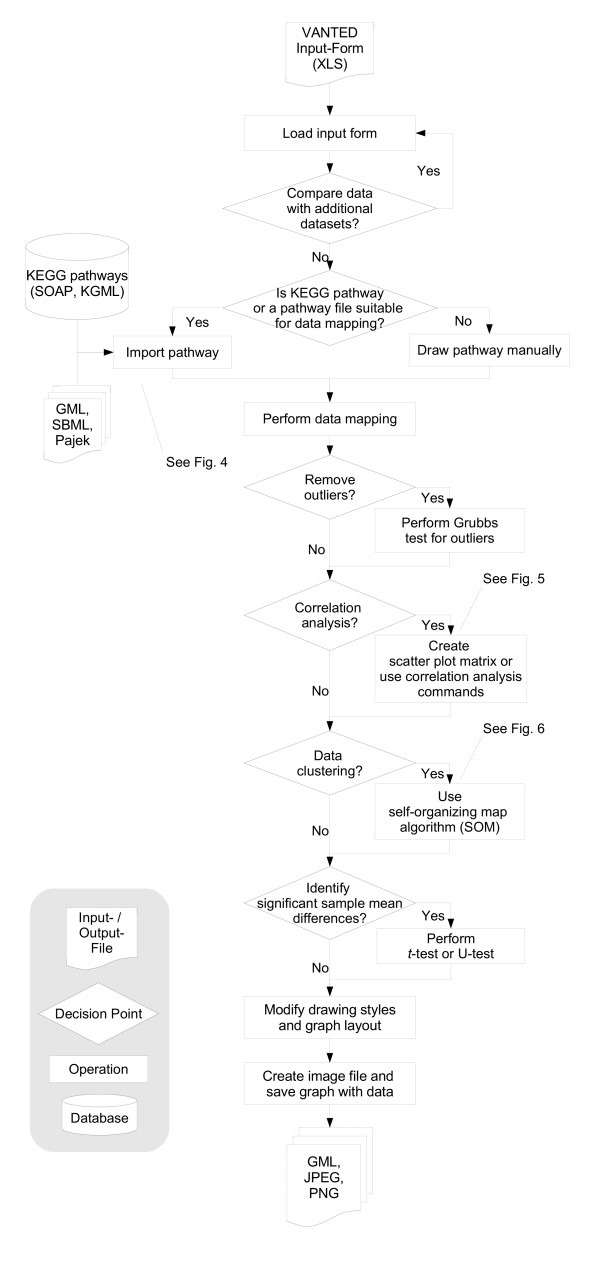
**Work-flow of a typical session in VANTED**. The pipeline for the visualization and analysis of biochemical data in the context of their underlying networks with VANTED. See Results section (Summary of VANTED's Features) for a detailed description.

**Figure 2:**
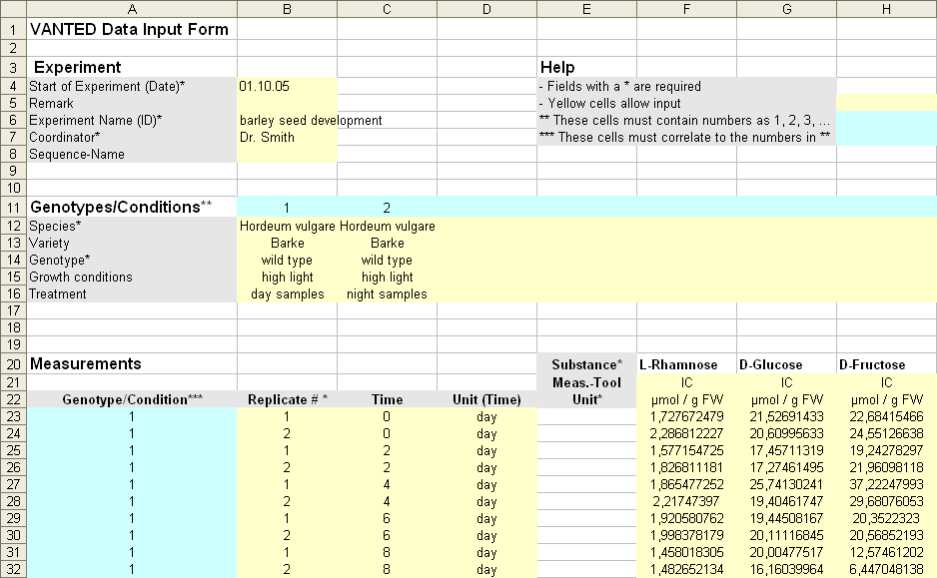
**Data input form**. The VANTED template may be saved and modified with all applications that support the Microsoft Excel file format, e.g. MS-Office Excel, Open Office and Gnumeric. Information about the experiment, a description of the genotypes or sample conditions, and finally the data values including replicate number, sample time, measuring tool, and unit may be filled in. The template is then imported into VANTED for data visualization and analysis.

**Figure 3:**
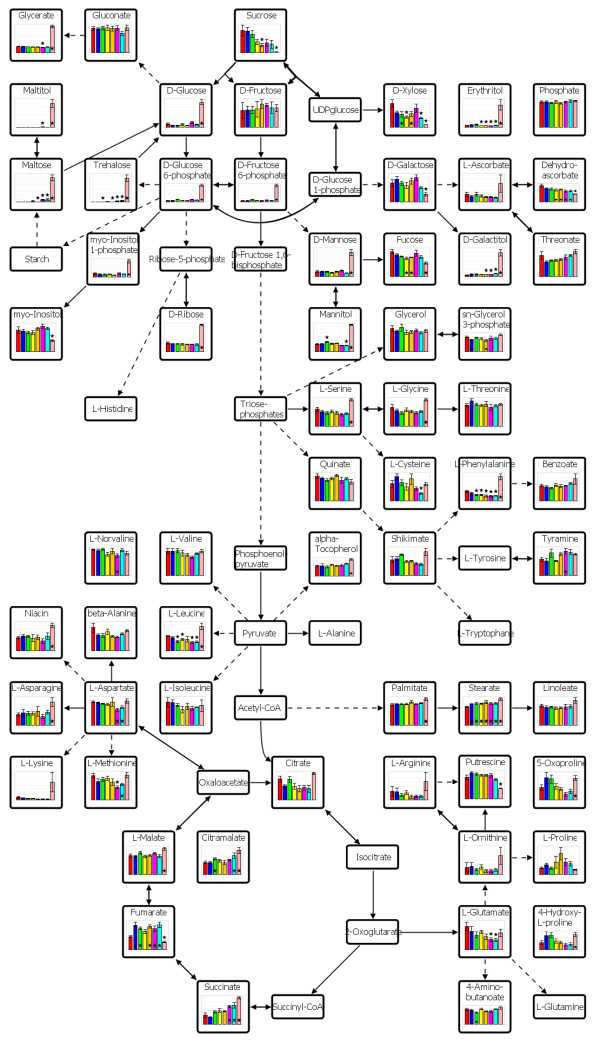
**Metabolite data mapped on plant central metabolism**. The metabolic network of the potato tuber was user-generated in VANTED. Metabolite profiling data of wildtype potato (*Solanum tuberosum*) tubers, and tubers expressing a yeast invertase either in an inducible or constitutive manner [33] was mapped onto the network. Each node represents a metabolite, connected by solid and dashed lines that represent single and lumped enzyme reactions, respectively. Data are means +/- standard error of the mean (SEM) of six independent plants. Values significantly different from the wildtype control as determined by an unpaired *t*-test (*p *< 005) were automatically marked with an asterisk. The bars in each diagram from left to right represent the values for the wildtype control (red), six inducible invertase lines, and one constitutive invertase line (light pink). The picture was created in VANTED and saved as a PNG file.

**Figure 4:**
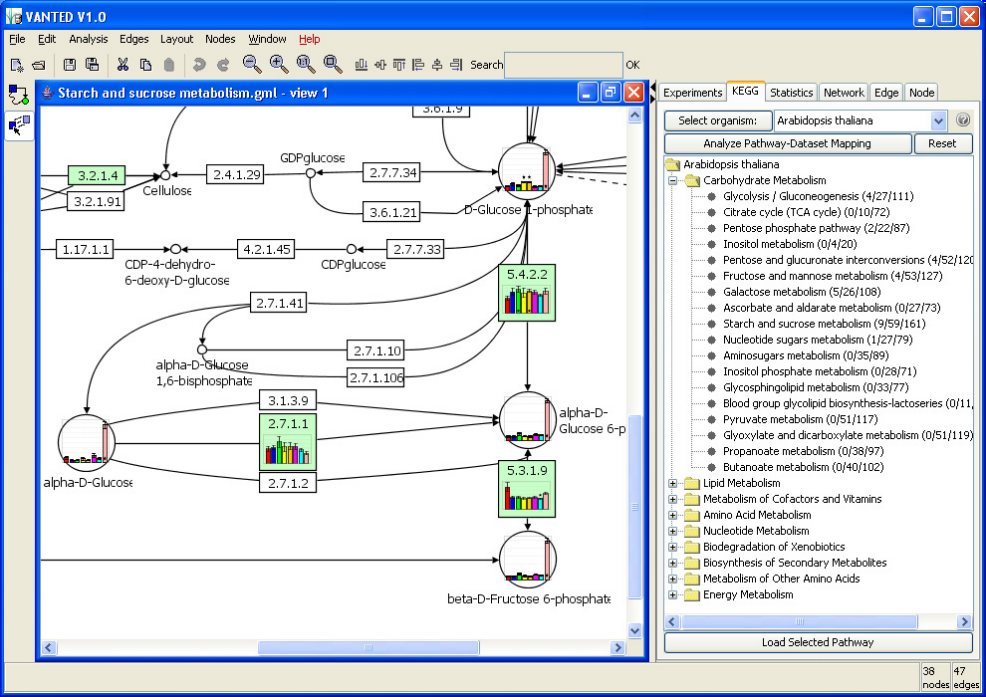
**Mapping of enzyme and metabolite data on KEGG pathways**. The pathway 500 (Starch and sucrose metabolism) was downloaded from the KEGG Pathway database [6]. Enzymes present in the model plant *Arabidopsis thaliana *as predicted from sequence information are shown in green. Selected enzyme activities and metabolite concentrations from wildtype potato tubers, and tubers expressing a yeast invertase either in an inducible or constitutive manner [33] were mapped on the pathway (which for better visualization was slightly modified using the built-in graph editor of VANTED). See legend of Figure 3 for details on the diagrams. The number of matches between the data set and all KEGG pathways is shown in the first number next to the pathway entry. The second number shows the number of enzymes in a pathway, the last the total number of nodes in the pathway (enzymes, metabolites, and links to other pathways).

**Figure 5:**
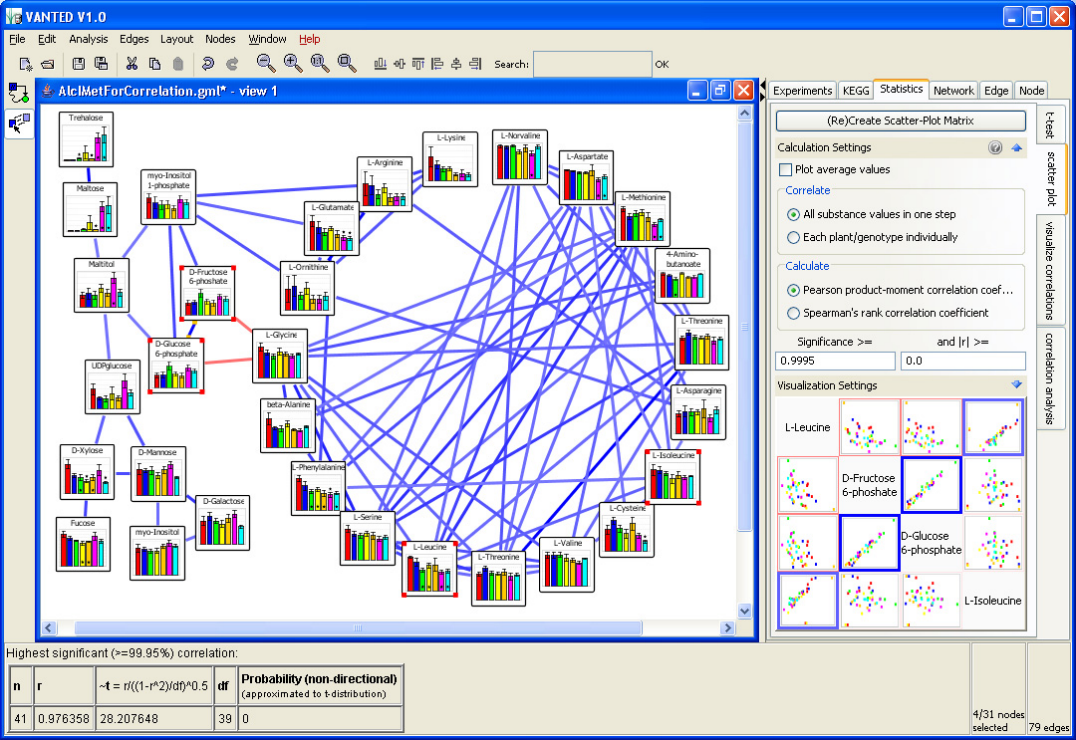
**Correlation graph and scatter plot generated from metabolite data**. Selected metabolite data (amino acids, sugars and sugar derivates) from potato tubers expressing a yeast invertase in an inducible manner [33] were mapped onto nodes before a correlation analysis was performed. See legend of Figure 3 for details on the diagrams. Positive and negative correlations are visualized by blue and red edges, respectively. The intensity of the edge depends on the value of Pearson's product-moment correlation coefficient. A combination of circular and force-directed layout was performed for better visualization. On the side-panel, a scatter plot is shown for the four metabolites that are marked with the small red squares in the network. Samples are color coded depending on the plant line. In the status bar, information is displayed concerning the correlation edge that is marked with yellow squares in the network (between D-Fructose 6-phosphate and D-Glucose 6-phosphate).

The work-flow of a typical session in VANTED is shown in Figure 1. Experimental data can be loaded into the system via a Microsoft Excel-based input form (Figure 2). This step can be repeated if additional data sets should be included for comparison. Depending on whether the KEGG database [6] contains a pathway that is suitable for the data mapping, (a) this pathway can be imported directly, (b) a network given in a standard network file format such as GML [25] or SBML [26] can be imported, or (c) a network can be created via a built-in graph editor and each node can be assigned to a metabolite, transcript or enzyme. The previously imported biochemical data can then be automatically mapped onto the nodes of the generated or imported network. The mapping creates a diagram for each node of the network for which experimental data is available. See Figure 3 for a user-created network and Figure 4 for a network imported from KEGG, both containing mapped data. If desired, the correlation between different substances can be calculated and visualized, either in the form of different node background colors if one substance should be correlated to the others, or in the form of new color-coded edges if all substances should be correlated against each other. The correlation can be either studied together with the network structure, or the original network can be removed as shown in Figure 5. Also, the substances can be clustered according to similar behavior over time using a self-organizing map (SOM) algorithm. To determine whether an observed difference of the sample mean is significant in comparison to the control data, different *t*-tests can be performed. If desired, automatic graph layout algorithms can be applied for better visualization of the network topology. Furthermore, changes can be made to the elements of the network with respect to diagram type, size, color, title, legend, and other characteristics. Finally, the resulting image can be stored as a GML file [25] if it should be edited later, or as a JPEG or PNG file for use in presentations or publications.

The graphical user interface of VANTED can be seen in Figures 4 and 5. The main display window is surrounded at the top by the main menu and a toolbar, at the left side by the buttons for graph editing, at the right side by a side panel allowing further activities for data analysis, and at the bottom by a status bar. In the following sections, we discuss the features and typical work-flow of VANTED in detail and then apply VANTED to two experimental data sets.

### Main functions of VANTED in detail

#### Data mapping and visualization

The data input of measurement values into VANTED is supported by an Excel input form (Figure 2). In addition to information about the general setup of an experiment, the form supports data from different -omics areas for different time points and for different genotypes or environmental conditions. This way the template acts as a single source for the input of multi-dimensional measurement data, which simplifies data handling.

VANTED allows the mapping of measurement data from different experiments onto arbitrary networks, which can be edited with the built-in graph editing functions. In addition to general graph editor functions such as node/edge selection, modification or deletion, an algorithm for the removal of node overlaps [27], and layout algorithms such as circular, tree-shaped and force-directed are available. As alternatives to network creation, networks may be loaded with the built-in importer from the KEGG Pathway database [6], or from the standard file formats GML [25], SBML [26], and Pajek .net [28].

The data mapping procedure will be done automatically if the substance name in the input form is equal to a target node label. The mapping procedure also considers any synonym or identifier defined in the KEGG Ligand database [29] or in the SIB (Swiss Institute of Bioinformatics) Enzyme nomenclature database [30]. If no automatic mapping is possible a user-defined mapping may be performed, in which a data subset for a measured substance needs to be assigned manually to a node. Additionally VANTED allows automatic creation of new nodes for all measurement data subsets which do not map onto the given network. The mapping of data onto (optionally organism specific) KEGG pathways is facilitated by a function which counts the number of possible automatic mappings of the measured substances onto the list of KEGG pathways (Figure 4).

The visualization of experimental data within the networks is done by including line or bar charts in the network nodes. Experimental data of different genotypes or plants may be shown within a single diagram inside each substance node, or shown in separate diagrams. The drawing style of the diagrams may be modified with a number of parameters such as series colors, the display of range or category labels, and line widths. As the system supports replicate measurement values in the data input form, the standard deviation (SD) or the standard error of the mean (SEM) may be shown as an error bar in both kinds of diagrams. For the line chart a polygon around the line may also be used to illustrate the variability of the data.

#### Computational data analysis

VANTED offers a variety of statistical functionalities for data analysis. At first, outliers in the data set may be identified and removed with the help of Grubb's test [23]. To compare experimental data from different plants or genotypes, a *t*-test can be used to determine whether the means of these data sets differ significantly or not. Depending on the assumption of equality of variances, Student's unpaired *t*-test or the Welch-Satterthwaite *t*-test can be carried out. Both *t*-tests assume the data to be normally distributed, that is, to follow a Gaussian distribution. This may be checked within VANTED with the David quick-test [23]. In the case that the data is not normally distributed a nonparametric rank-sum test (*U *-test) should be performed instead of a *t*-test.

Relationships or common patterns in the data can be found by plotting the measurement values for a defined set of substances inside a scatter plot matrix (Figure 5, right side). This matrix displays the measurement values for all combinations of the selected substance nodes. The Pearson correlation coefficient is visualized with a color-coded diagram frame (Figure 5, right side). In case of a significant correlation, the border width of the diagram is increased. Alternatively, Spearman's rank order correlation coefficient may be used, which is more robust against outliers. For the interactive analysis a reference node is selected and the correlation with all remaining nodes is visualized by different node colors. Significant correlations are again highlighted by an increased border width. A gamma correction may be used to emphasize strong correlations.

To create a correlation network from a number of selected substance nodes, the correlation between all possible pairs of nodes can be calculated, and a new edge is created between two nodes if this correlation is significant (Figure 5, left side). Different edge colors are used to visualize positive and negative correlations. A built-in force-directed graph layout algorithm may now be used to visually group significantly correlated graph nodes.

**Figure 6:**
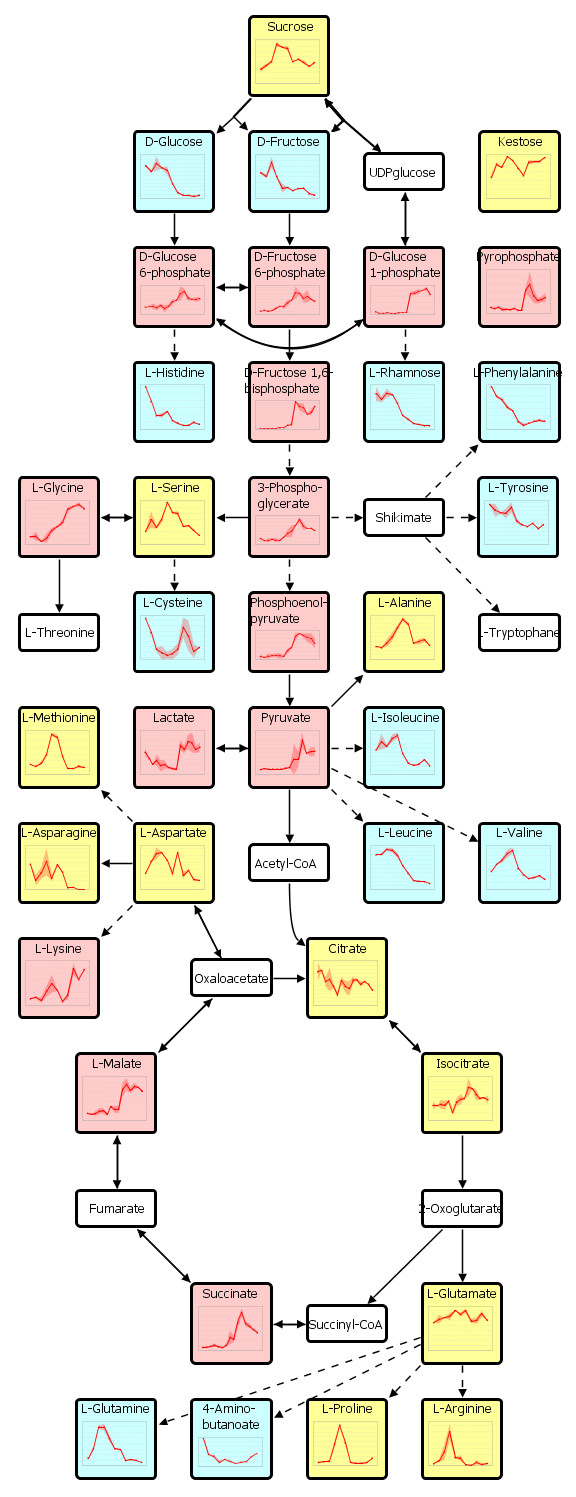
**Time series data clustered by self-organizing maps**. Wildtype barley (*Hordeum vulgare*) caryopses were harvested every second day over a growth period of about 20 days post anthesis and analyzed for the dynamical changes of several central metabolites. The data set was mapped onto a network that was previously created in VANTED. Each node represents a metabolite, connected by solid and dashed lines that represent single and lumped enzyme reactions, respectively. Data are means of two independent plants, the standard error of the mean (SEM) is shown as a polygon around the line. A self-organizing map algorithm was performed to cluster the metabolites into three groups by similar behavior over time, which is visualized by the background color.

In addition to the statistical functions a neuronal network algorithm (a self-organizing map, SOM) is included in the system. The SOM is a powerful method for visualization and classification tasks. It has been used for speech recognition, robotics, process control [24], and to cluster gene expression data [31]. In VANTED the SOM is used as a tool for the extraction of common measurement patterns over time. At first a training phase of the SOM needs to be performed. During this phase the SOM adapts itself to common input patterns, a process that can be influenced by various parameters. Subsequently all nodes are assigned to clusters, based on the best matching SOM node. As a result the substances are grouped according to similar patterns. These clusters can be color-coded (Figure 6). Similar patterns are easily discovered visually even if they are widely spread over the picture.

### Experimental case studies

#### Metabolite and enzyme data from genetically modified potato tubers

The regulation of sucrose to starch conversion in the potato tuber has been extensively studied in the last few decades (for a review see [32]). In an attempt to better understand the importance of sucrose mobilization in this pathway, a yeast invertase was expressed in an inducible manner in growing potato tubers [33], and the metabolite changes were monitored by a metabolite profiling approach coupling mass spectrometry to gas chromatography [1]. This method allows the measurement of the relative concentrations of more than 60 metabolites simultaneously. The resulting mid-scale data sets have to date mostly been presented in complex tables [1,33]. With VANTED, the values can now be analyzed in the context of the underlying pathways, which allows a more comprehensive picture of the processes taking place in metabolism upon transgene expression.

In Figure 3, a metabolic network of plant central metabolism that was drawn using the graph editor function of VANTED is shown. The example data set that was mapped on the nodes consists of 62 relative metabolite concentrations from developing tubers of eight potato genotypes (one wildtype, one constitutive and six inducible yeast invertase lines), each from six replicates, giving a total number of close to 3000 values [33]. The network was drawn especially for this data set, however it can be adapted to any other data set from central metabolism, depending on which substances were measured and are to be displayed. For a better understanding of the pathways, some metabolites that have not been measured were additionally included. With this visualization it becomes evident that some metabolites and even whole sections of the displayed part of plant metabolism seem to be coupled, while others show different behavior. For example, some, but not all, carbohydrates are massively increased upon expression of the constitutively expressed yeast invertase, while they do not show large changes upon an inducible expression of the same enzyme. The intermediates of the citrate cycle are only in some cases significantly increased, while it is known that invertase expression leads to a large increase in the flux through glycolysis [34]. Finding these coherences from a large table of numbers would require comprehensive knowledge and the capability to intuitively handle the metabolic maps, which is of course desirable, but not always possible, for researchers. 

For a subset of this metabolite data set consisting of amino acids, sugars, and sugar derivatives, a correlation network has been generated with VANTED (Figure 5). The strongest correlations were observed between glucose 6-phosphate and fructose 6-phosphate, and between leucine and isoleucine, which can also be seen from the scatter plot matrix shown on the right side of Figure 5. This observation is in accordance with a previous study in which these correlations were shown to be the strongest ones in a different data set [1]. From the network image it can be seen that the amino acids form a highly connected cluster, the sugars and sugar derivatives form a loosely connected cluster, and there are only a few links between these clusters: a negative correlation between hexose phosphates and glycine, and positive correlations between inositol 1-phosphate and the amino acids glutamate, arginine and ornithine. These findings are consistent with another study in which it has been shown that glucose and mannitol are negatively correlated to a highly connected amino acid cluster [35].

In the study mentioned above, in addition to metabolite levels the activities of several glycolytic enzymes were also measured [33]. For the image shown in the main display window in Figure 4, a subset of the original data was mapped onto map 500 (starch and sucrose metabolism) from the KEGG Pathway database. One goal in the development of the KEGG import was to achieve an appearance similar to the KEGG pictures. Now, in contrast to the static KEGG pictures, the network can be further edited by the user. In the data visualization shown in Figure 4 it is immediately visible that the constitutive expression of the yeast invertase leads to massive increases in hexoses and hexose phosphates, while the activity of the corresponding enzymes are not significantly altered. A reason for this might be that the corresponding enzyme levels are high enough to cope with temporally, but not constant, increases in hexose levels.

#### Time series metabolite data from developing barley seeds

Cereal seeds accumulate starch and proteins as storage products. Despite extensive studies on the biochemistry of cereal seeds [36], the regulatory mechanisms underlying their high storage capacity remain largely unknown. In an attempt to elucidate the control of the cell's energy state on starch accumulation, a large data set was created containing the dynamic changes of about 40 metabolite concentrations determined from barley caryopses (*Hordeum vulgare*) in the middle of every second day over a growth period of about 20 days post anthesis [37]. The network edited for Figure 3 was modified to be appropriate for the mapping of this data set (Figure 6). After data mapping, a self-organizing map [24] with 6 neurons was trained using all nodes to find similar patterns in the behavior of the metabolites over time. From the resulting 6 cluster prototypes, 3 clusters have been created that show (a) a decrease or (b) an increase over time, or (c) either high levels in the middle of the time frame or no significant pattern. The three different clusters were then automatically visualized by three different node colors (Figure 6). It can be seen that metabolites close together in a pathway tend to fall into the same group, as for example hexose phosphates and glycolytic intermediates all belong to the cluster in which the concentration increases over time, which is in accordance to the observation that glycolytic genes are also induced at the onset of storage [38].

## Discussion

The increasing size of data sets generated in biological research creates a strong need for automatized data analysis and visualization tools. Therefore we designed VANTED, a platform-independent tool that allows the visual analysis of mid- and large-scale biochemical data sets in the context of relevant networks. In the following we will describe other existing tools in comparison to VANTED.

There are a number of tools which facilitate the editing and visualization of biological networks, among them BioMiner [8], PaVESy [9], VisANT [39], Patika [7], and Osprey [40]. In most cases standard layout methods such as force-directed [41] and hierarchical layouts [42] are used to visualize biological networks. Patika extends the force-directed layout to deal with application specific requirements in biological research, especially for cellular compartmentation. Osprey allows manipulation and visualization of interaction networks and supports search and filter operations. BioMiner and PaVESy are both equipped with an internal pathway database. BioMiner facilitates finding possible paths from one metabolite to another. PaVESy uses the network analysis toolkit Pajek [28] to visualize the pathways. The VANTED system presented in this paper supports several layout algorithms and thus can be also used as a tool for the editing and visualization of biological networks.

All the general visualization tools mentioned above are usually restricted to visualization and manipulation of the biological network, and thus do not support mapping and visualization of experimental data. For this task, there are a number of tools available which display data on static or dynamic networks with the focus on gene expression data. Probably the most prominent example is the Omics Viewer which allows large scale data from experiments such as microarray expression profiling, proteomics, and metabolic profiling to be overlaid onto a metabolic map from the MetaCyc databases [14], e. g. AraCyc [15].  Concentration differences are shown by color gradients (also called heatmaps). Multiple experiments can be compared in that the different colored maps are displayed one after the other in an animation. However, in contrast to VANTED, with this tool it is neither possible to edit the network, nor to visualize multiple data sets in a single image.

Similar to the Omics Viewer, many other tools are linked to databases. In the case of MetNet [43] and ToPNet [44], the database is included in the tool itself, but only ToPNet also allows editing and layout of the pathways. MapMan [11] and KappaView [12] both rely on libraries of pathways that are stored in the tool as pictures. This strategy, however, is of limited use because the pathways can not be edited and layouted dynamically. Instead the user needs to modify the picture manually and inform the tool about the new position of the nodes. It should be noted that the main intention of these two projects is the annotation of the genes present in the expression profiles to functional groups, and not the provision of a computational framework for mapping data onto general networks. In other tools such as PathwayExplorer [13], PathMAPA [45] (and its successor VitaPad [46]), and PathwayAnalyser in the PathwayProcessor software package [47] the pathways are loaded from the KEGG Pathway database [6], a procedure that is also available in VANTED. PathwayExplorer [13] is a versatile web-based tool that also makes it possible to display time series expression data in the enzyme nodes of KEGG pathway maps by applying color gradients to stripes of the node, but it is neither possible to dynamically modify the pathway maps, nor does it support the mapping of metabolite data.

Nearly all of these data visualization tools allow the display of gene expression data on the network, but only the Omics Viewer [15], Cytoscape [10], and MapMan [11] are designed to also display metabolite or other data. With the exception of MetNet [43] and, as mentioned before, Omics Viewer and PathwayExplorer, all of the tools are limited in that they only allow the display of the data from two experiments in comparison as a color code (heatmap). However, MetNet groups the data into pathways and is thus not able to display the data of single enzymes on the pathway structure. Hence, to our knowledge, VANTED is the first tool to display -omics data from multiple experiments superimposed on a network in one picture.

Only a few tools for data visualization also include statistical analysis. For example, with PathMAPA [45] and PathwayExplorer [13] it is possible to perform Fisher's exact test to determine whether the expression of the genes in a given pathway is affected by a specific experiment. To our knowledge, in contrast to VANTED no other data visualization tool allows direct determination of correlations within the data set, construction of correlation networks from these, and clustering of data with machine learning methods such as self-organizing maps.

In particular the creation of correlation networks from biochemical data is currently of great interest. Several recent studies have shown that valuable additional information can be derived from large-scale transcript [48,49] and metabolite [35,50] data sets. As VANTED provides researchers with the possibility to generate and visualize correlation networks in the context of theoretical pathway networks, it is a valuable tool to support these recent developments.

One limitation of VANTED is that with the current version it is not practicable to visualize genome-wide data sets. Typically analyzed data sets should contain up to a few hundred items (metabolite, enzymes) from up to a few dozen conditions (or genotypes, or time points) with a large number of replicates. However, it has to be noted that VANTED offers great flexibility. There is no limitation on the type of networks and data: the networks for example could also consist of tree-like hierarchical structures such as the MapMan Bins [11], and instead of enzyme activity data as shown in this study it is possible to display gene expression data.

With the experimental case studies, we have shown that with VANTED it is possible in relatively short time to find previously known relationships between substances, and to observe new relationships.

### Future perspectives

VANTED is a state-of-the-art tool for the visual analysis of biological data in the context of relevant networks. After making it publicly available for academic use, we anticipate that it will find wide acceptance in the scientific community. We are collaborating closely with researchers to improve VANTED, especially to add new functionalities depending on user needs. Furthermore, any comments and suggestions from the research community are greatly appreciated. In the future we are planning to offer a heatmap functionality and the possibility of hierarchical network representation to allow the visualization of genome-wide data sets.

## Conclusion

We have developed VANTED, a platform independent tool, available free of charge to the scientific community. It helps scientists to interpret their biochemical data sets by analyzing and visualizing them in the context of the underlying metabolic pathways or other networks. A large number of data visualization tools for various purposes have been created in the last few years, but VANTED is unique in its combination of numerous features of which other data visualization tools provide only a subset: dynamic network editing and layout, mapping of medium- to large-scale experimental data sets from different time points or conditions on networks, statistical tests, generation of correlation networks, and clustering of similarly behaving substances. Furthermore, it allows the display of data in a so far unequaled level of detail. These features in combination with the simple and intuitive graphical user interface should make VANTED a valuable tool for a broad range of researchers.

## Availability and requirements

• **Project name: **VANTED

• **Project home page: **

• **Operating system(s): **Platform independent

• **Programming language: **Java

• **Other requirements: **Java version 1.5 or higher, screen resolution of 1024 × 768 or higher, mouse, 512 MB RAM recommended

• **License: **VANTED is available free of charge.

• **Any restrictions to use by non-academics: **Commercial users need to adhere to the KEGG license terms in case the KEGG related functions are used.

## Authors' contributions

CK and FS designed the architecture of the system. CK implemented the system. All three authors evaluated the system and participated in its final design. BHJ contributed the experimental case studies. BHJ and CK drafted the manuscript. All authors read, revised and approved the manuscript and are listed in alphabetical order.
